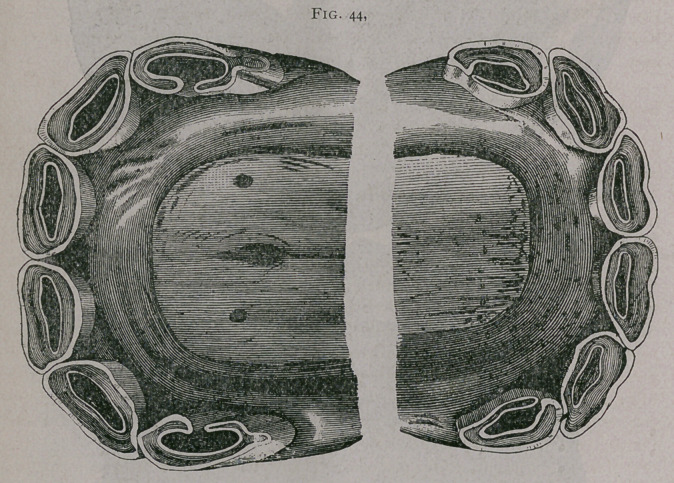# Age of the Horse, Ox, Dog, and Other Domesticated Animals

**Published:** 1890-10

**Authors:** R. S. Huidekoper

**Affiliations:** Veterinarian


### AGE OF THE HORSE, OX, DOG, AND OTHER DOMESTICATED ANIMALS.

BY R. S. HUIDEKOPER, M.D., VETERINARIAN.

*Continued from page 509.*

FOURTH PERIOD.—Leveling of the permanent incisors. During this period the signs furnished by the dental apparatus become more difficult to recognize, and the determination of the exact age is less precise than at an earlier period. The points to be examined from six years are, first, the wearing of the corner teeth, the form of the transverse diameters of the teeth, the position of the central enamel on the surface of the table and the general outline of the incisive arch.

FIG. 43.

*Six Years, Fig. 43.*—In front the teeth appear much as they were at five years of age. In profile we see in this animal a more tardyeruption of the tush teeth, which are not yet quite free from the gums, and are, therefore, of little value as regards the age. The tables furnish a most accurate guide; the posterior border of both inferior and superior corner teeth are worn; the pincher teeth are leveled and their tables tend to an oval form. It is seen, however,

FIG. 43.

that the inferior cups are thicker at their anterior border, due to a small portion of the surface enamel still remaining. The cups are narrower from side to side than at five years and somewhat closer to the posterior border of the table; the same appears on the intermediate teeth. It will be noticed that the cups of the superior corner teeth have fissures on their posterior borders which is of frequent occurence and does not interfere with judging the amount of work which they have performed.

FIG. 44.

*Seven Years.*—There is nothing special to be seen in front except that the teeth are whiter, due to the disappearance of the cement which has been worn from the surface of the enamel. In profile it is seen that the table of the inferior corner tooth is narrower than that of the superior from the front to behind, so that a notch is formed on the posterior corner of the latter. The incident of the tooth is less perpendicular than at six years. The

FIG. 44.

cups of the table of the intermediate teeth are wider from in front to behind and narrower from side to side. In the corner teeth the wearing surface is larger and the cups are smaller. The pincher teeth are oval and the intermediate teeth commence to become so. In this mouth, again, the superior corner teeth are fissured on their posterior border.

(TO BE CONTINUED.)

## Figures and Tables

**Fig. 43. f1:**
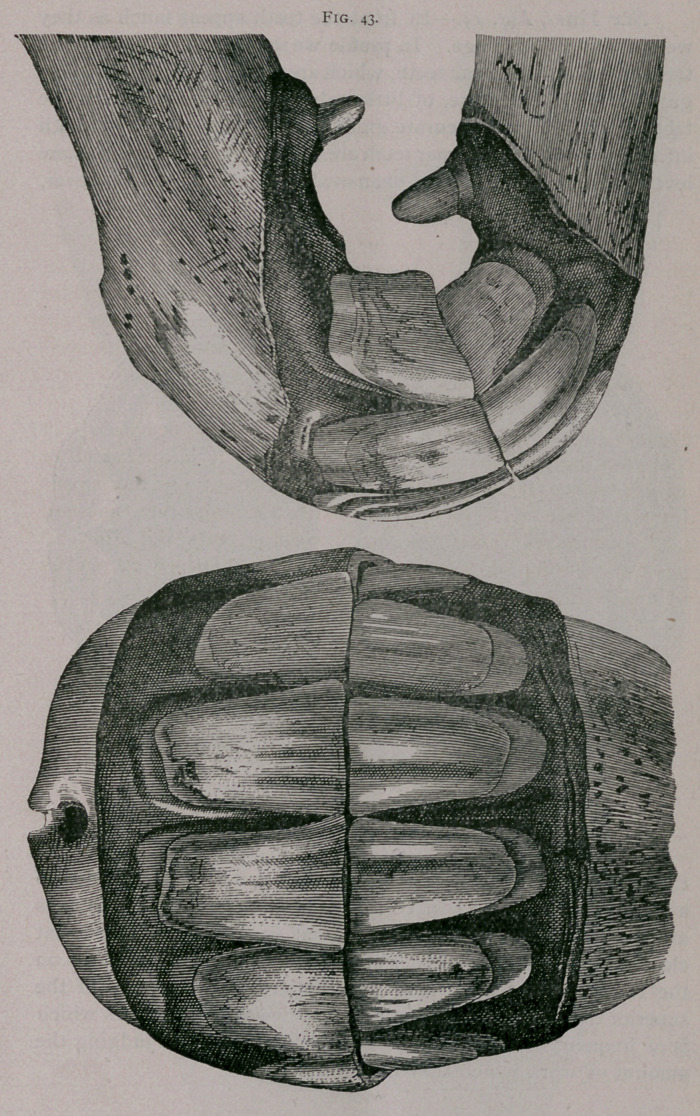


**Fig. 43. f2:**
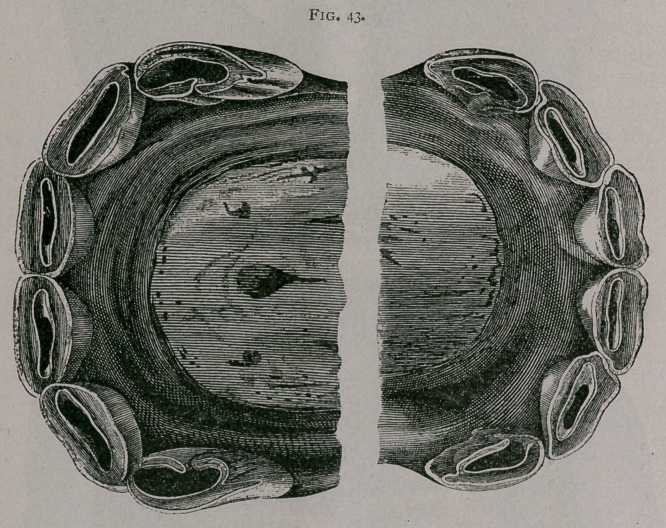


**Fig. 44. f3:**
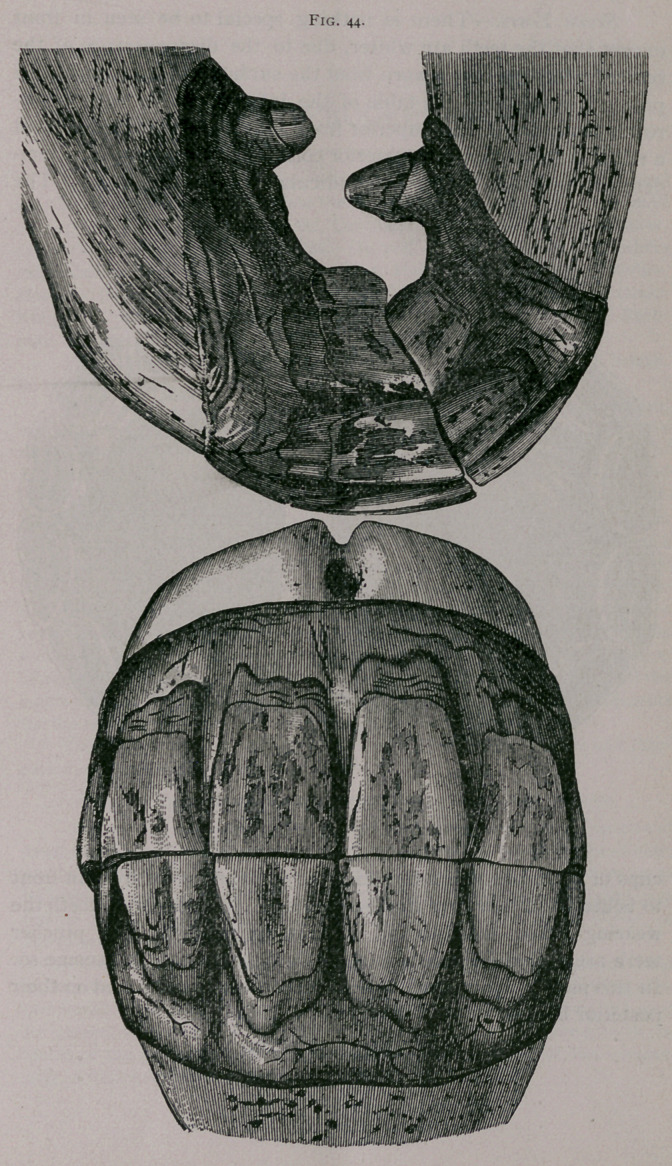


**Fig. 44. f4:**